# The ‘10 Excess’ Phenomenon in Responses to Survey Questions on Happiness

**DOI:** 10.1007/s11205-016-1265-x

**Published:** 2016-03-30

**Authors:** Gaël Brulé, Ruut Veenhoven

**Affiliations:** 1 Erasmus Happiness Economics Research Organization, Erasmus University Rotterdam, POB 1738, 3000DR Rotterdam, The Netherlands; 2 North West University, Vanderbijlpark, South Africa

**Keywords:** Life satisfaction, Happiness, 10 excess, Cultural effect, Extreme responding

## Abstract

Happiness in nations is typically measured in surveys using a single question. A common question is: ‘all things considered, how satisfied or dissatisfied are you with your life as-a-whole these days on a scale from 0 to 10?’. The responses typically follow a uni-modal distribution with highest frequencies between 5 and 8. Yet in some nations, the percentage of 10 responses stands out and is higher than the percentage of 9 responses. This is particularly present in Latin America and in the Middle East. In this paper we explore the prevalence of the ‘10-excess’ pattern and check some possible explanations. We conclude that the 10-excess phenomenon is partly due to cultural influence.

## Introduction

Happiness became a subject of empirical social science research in the second half of the twentieth century. To date (2015) some 3500 empirical studies have been carried out on happiness, most of which are recorded in the Bibliography of Happiness, part of the World Database of Happiness (Veenhoven 2015). Of these 3500 empirical studies, approximately 600 compare nations. Landmark studies such as these have been conducted by Cantril (1965), Inglehart (1977, 2000) and Diener et al. (2010).

Survey research involves questioning, typically using ‘closed’ questions. For instance, respondents are presented with a standard question and answer by choosing one of a few response options, such as ‘very happy’, ‘pretty happy’ or ‘not too happy’. Questions of this type are presented in personal interviews, in questionnaires or via the Internet. Each method of collecting information can be biased in various ways. Additionally, responses to survey questions may fail to measure what they are supposed to. In this regard, Bourdieu (1994) argues that closed questions might shed light on topics that people would not otherwise consider. Likewise, Morin (1994) argues closed questions ‘trap’ respondents in pre-established schemata. An objection particular to survey questions on happiness is that such questions tap into how happy respondents feel they should be given their situation, rather than how happy they actually are. These qualms have given rise to many validity tests, see the 31 publications listed in section Ca01 of the Bibliography of Happiness (Veenhoven 2015). The conclusion is that the validity of such responses is viable, provided that questions clearly address subjective appreciation with one’s life as a whole. Still there are persistent qualms about the reliability of answers to questions about happiness.

Responses to questions about happiness might reflect the respondents’ life satisfaction, but may do so inaccurately. Responses can be distorted in several ways. It has been suggested that *desirability* *bias* produces unrealistically high scores on happiness; for instance self-ratings of happiness tend to be slightly higher in personal interviews than on anonymous questionnaires (Phillips and Clancy 1972). An *interviewer* *bias* occurs when responses are influenced by the interviewer’s characteristics; for instance, if the interviewer is in a wheelchair, the benefit of good health is salient. Respondents in good health then rate their happiness somewhat higher and the correlation of happiness-ratings with health variables is more pronounced (Smit et al. 1995). *Extreme* *response* *bias* (*ERB*) characterizes the tendency of some respondents to tick the highest or lowest option. Greenleaf (1992) found that this tendency is related to the age, education level, and household income of respondents, but not to their gender. Diener et al. (1991) report that mood intensity is quite vulnerable to ERB. Maggino and Schifini D’Andrea (2003) found that longer scales are less exposed to ERBs than shorter scales. *Contextual* *biases*, such as the presentation of the study, the conversational context (Smith et al. 2006) and the day of the week can also influence the response of interviewees. For instance, responses to questions about satisfaction with one’s life as a whole tend to be slightly more positive when asked on a Monday than on a Friday, Saturday or Sunday (Akay and Martinsson 2009). Finally, *questionnaire* *effects* can also have undesired effects. For instance, the order of questions has been proved to influence both the distribution of responses and association with other variables, e.g. the observed correlation between happiness and income tends to be higher if the question on happiness follows after questions about income (Glenn and Taylor 1990).

The various biases mentioned above can be random or systematic. A random bias is no great problem in cross-national happiness research, since random distortions typically balance out in big samples. A systematic bias is trickier, in particular when cultural factors are involved. This is called a *cultural* *measurement* *bias*. In this context the ERB deserves consideration, as this kind of bias appears to differ across cultures. Culpepper and Zimmerman (2006) have shown in a study conducted in an American university that Hispanic students were more prone to extreme responses. Hispanic students were less likely to go for middle responses and would go more for extremes than their Anglo-Saxon counterparts. Likewise, Chinese students were less inclined to extreme responses than Caucasian students (Song et al. 2011). In a bi-ethnic comparison in Israel, Arab respondents have been shown to veer more towards extreme responses than their Jewish counterparts (Baron-Epel et al. 2010). In this respect, one can question the attempt of Johnson et al. (2005) to link response styles with Hofstede’s measures, as those are themselves also heavily dependent upon response styles.

**Fig. 1:**
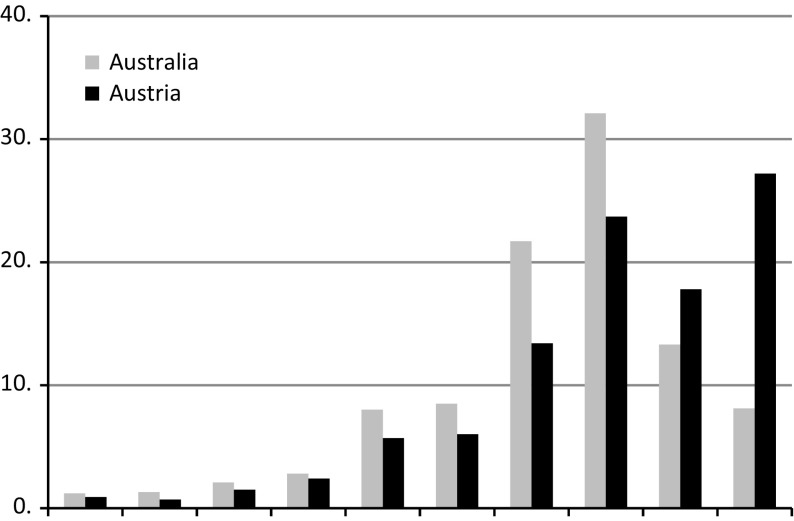
Two distributions of responses to a single question about life satisfaction. Classic, Australia 2005 and 10 excess, Austria 1999

A particular type of ERB appears in responses to survey questions about happiness. When using a numerical response scale ranging from 0 to 10, the percentage of responses in the highest category (10) is surprisingly high in several countries and sometimes, option ‘10’ is ticked more often than option ‘9’. One consequence is that the overall distributions no longer fit the unimodal distribution often observed, in particular in developed nations. An example of such a frequency distribution is presented in Fig. 1. Such is the case in Austria, where the percentage of ten responses is almost twice as high as the percentage of nine responses, contrary to Australia, which shows a more classic unimodal curve. We call this the *10* *excess* phenomenon. As we will see in more detail in Sect. 4, this pattern appears in many countries all over the world and is particularly frequent in Latin America and the Middle East.

The goal of this paper is to explore the 10 excess phenomenon. More specifically, we want to answer the following questions for the first time: How often does it appear and where? What are the possible reasons behind this response pattern? This is important to know as the 0–10 numerical response scale has become standardized in happiness studies. In that respect, the more we know about the various biases, the more they can be taken into account in future studies. We will first describe in more detail what we mean by ‘happiness’, how it is measured and how we define ‘excess’ (Sect. 2). Next we will assess the prevalence of the 10 excess phenomenon, how often it occurs and in which nations in particular (Sect. 3). On that basis we suggest several possible explanations for this phenomenon and verify these one by one (Sect. 4). Most of the explanations we considered failed an empirical test. We conclude that some cultural effect is likely to be involved (Sect. 5).

## Subject Matter

In philosophy, the term ‘happiness’ often denotes the good life in a broad sense. In contemporary empirical research, the term is mostly used for subjective satisfaction with life. In this tradition, Veenhoven (1984) defines happiness as *the* *degree* *to* *which* *someone* *evaluates* *the* *overall* *quality* *of* *his* *or* *her* *present* *life*-*as*-*a*-*whole* *positively*. In other words, how much one likes the life one lives. Thus defined, happiness is something people have in mind, and happiness in this sense can therefore be measured with questionnaires. As such, happiness is a suitable subject for survey research. The survey question on happiness under consideration in this paper reads as follows: “Taken all together, how satisfied or dissatisfied are you with your life-as-a-whole these days? Please answer by ticking a number between 0 (or 1) and 10, where 0 (or 1) stands for dissatisfied and 10 for satisfied”.

This kind of question has been replicated in several large scale survey studies, many of which are part of an international survey program, such as the Gallup World Poll, the World Values Survey, the European Social Survey, the Eurobarometer and the Latino Barometro. The observed distribution of responses in each of these studies are gathered have been collated in the collection of ‘Happiness in Nations’ (Veenhoven 2015), in which they have been categorized by question type, defined by (1) the keyword used in the question, e.g. ‘happiness’, (2) the time frame addressed, e.g. ‘these days’, (3) the response scale, verbal or numerical and (4) the number of response options.

**Fig. 2:**
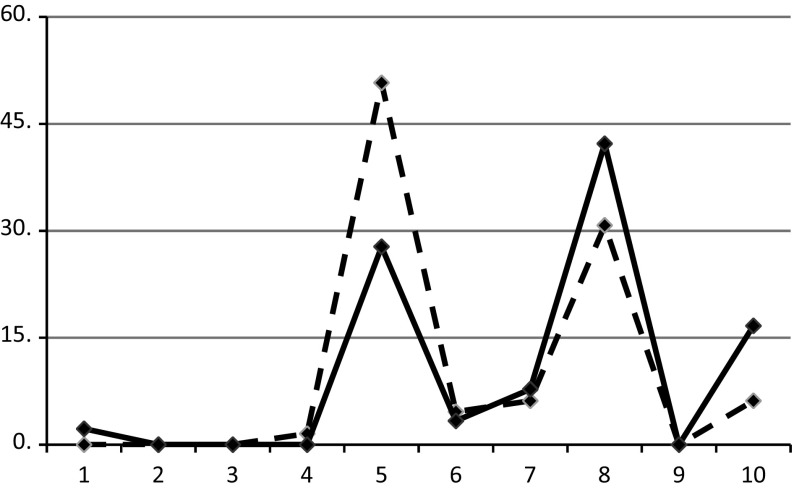
Distribution of the highest values for the latest data on a 0–10 scale (dashed line, 65 nations) and on a 1–10 scale (plain line, 90 nations)

We observed this collection of data responses and considered the findings obtained for questions based on a numerical response scale (type n), with at least 10 options, that is, either 1–10 scales, as in Fig. 2, or 0–10 scales. This type of response scale has been used for various keywords such as ‘happiness’, ‘life-satisfaction’ or ‘best-worst possible life’, a rating which is better known as the Cantril ladder (Cantril 1965).

To date (2015) these kinds of questions appear in more than 5000 survey studies in the general public of nations and in 1367 of the surveys the full distribution of responses have been recorded, rather than only the mean and standard deviation. In this paper, we observe the 10 excess responses in the 1367 distributions (Sect. 3). The number and type of surveys are presented in Table 1, in which elementary statistics are given. They represent all the distributions present in the World Database of Happiness.

**Table 1:** Descriptive statistics of the distributions

Survey name		Number of surveys	Years	N
*Distribution 1–10*				
World values survey		750	1981–2005	133–3000
EQLS		59	2003	600–1000
Other surveys		24	1982–2006	424–2400
TOTAL		833	1981–2006	133–3000
*Distribution 0–10*				
ESS		224	2002–2006	579–3027
PEW		52	2005–2007	500–3142
Gallup		47	1979–2011	495–5000
LAPOP		45	2008–2010	1171–2951
What World Thinks		50	2002	500–2032
Other surveys		116	1980–2009	642–2986
TOTAL		534	1979–2011	495–5000

We speak of ‘10 excess’ when the number of respondents answering ‘10’ is higher than the number of respondents answering ‘9’. The degree of excess is expressed in a Ten Over Nine ratio, that we call the TON. We speak of 10 excess when TON is greater than 1.

## Prevalence of 10 Excess

Out of the 1367 distributions previously mentioned, 534 are on a numerical scale ranging from 0 to 10 and 833 on a scale ranging from 1 to 10. Since this small variation in scale length might induce a difference, we considered them separately.^1^ Before presenting the frequency of appearance of the 10 excess for the different scales, we observed the distribution of responses, and presented an illustrative overview of the most recent scores on the 1–10 scale in 15 nations in Table 2.

**Table 2:** Distribution of responses to a question on life-satisfaction in the years 2006–2009 on a *1*–*10* numerical scale

	1	2	3	4	5	6	7	8	9	10	TON ratio
Albania	4.6	6	15.4	15.9	16.7	11.7	11.3	10.2	5.3	2.7	0.51
**Algeria**	12.6	5.6	8.4	5.9	12.9	10.5	13.6	11.5	6.3	11.9	**1.89**
Andorra	0.5	0.4	1.3	2.5	12.5	13.3	23.1	29.7	9.9	6.7	0.68
**Argentina**	1.2	0.7	2	1.4	7.3	5.3	18.8	26.6	13.4	22.5	**1.68**
Armenia	14.3	10.5	16.5	12.8	17	9.3	8.2	5.7	2.9	2.5	0.86
Australia	1.2	1.3	2.1	2.8	8	8.5	21.7	32.1	13.3	8.1	0.61
**Austria**	0.9	0.7	1.5	2.4	5.7	6	13.4	23.7	17.8	27.2	**1.53**
**Bangladesh**	3.3	1.1	7.5	10.5	35	9.3	8.9	10.6	2.9	9.5	**3.28**
Belarus	6.8	8.3	14.2	13.5	21.5	8.9	10.1	8.1	3.3	1.9	0.58
**Belgium**	3	0.7	1.7	2.6	6.3	7	15.9	29.4	15.7	16.8	**1.07**
**Bosnia**	7.3	3.3	5.1	8.6	24.3	13.3	13.2	11.6	5.3	7.8	**1.47**
**Brazil**	1.9	0.9	1.4	2.2	10.9	8.6	12.4	23.8	13.4	24.3	**1.81**
Bulgaria	5.8	6.6	11.3	11.6	18.7	11	14.9	8	4.8	2.8	0.58
**Burkina**	3.6	4.6	6.7	12.1	26	15.2	11.1	8.2	3.1	7	**2.26**
Canada	0.8	1.2	1.4	2.6	6	7.3	14.1	28.6	19.4	18.6	0.96

First 15 cases out of 90

Data: Happiness in Nations (Veenhoven 2015), Table 122F

Countries in bold are illustrative of the 10 excess phenomenon

We then assessed the frequency of the 10 excess in all 1367 surveys that have included a question on life satisfaction. The frequency on a 1–10 scale is reported in Table 3 and the frequency on the 0–10 scale in Table 4. We also assessed the frequency of the pattern in questions about contentment, see Table 5.

**Table 3:** 10 excess frequency in responses to a question on life satisfaction on a *1*–*10* numerical scale

	Surveys with TON>1	Total number of surveys	Ratio
Albania	0	6	0.00
Algeria	4	6	0.67
Andorra	0	4	0.00
**Argentina**	**11**	**11**	**1.00**
Armenia	4	8	0.50
Australia	1	9	0.11
Austria	3	4	0.75
Azerbaijan	2	9	0.22
Bangladesh	11	14	0.79
Belarus	3	10	0.30
Belgium	2	6	0.33
**Bosnia Herzegovina**	**4**	**4**	**1.00**
**Brazil**	**10**	**10**	**1.00**
Bulgaria	4	16	0.25
Burkina Faso	6	9	0.67
**All 97 cases**	**462**		**0.54**

First 15 cases out of 97

Data: Happiness in Nations (Veenhoven 2015), Table 122F

Countries in bold are illustrative of the 10 excess phenomenon

**Table 4:** 10 excess frequency in responses to a question on life satisfaction on a *0*–*10* numerical scale

	Number of surveys with TON>1	Total number of surveys	Ratio
Angola	0	1	0.00
Argentina	1	5	0.20
Australia	4	6	0.67
Austria	3	6	0.50
Bangladesh	0	2	0.00
Belgium	1	14	0.07
**Belize**	**1**	**1**	**1.00**
Bhutan	7	22	0.32
Bolivia	2	5	0.40
**Brazil**	**7**	**7**	**1.00**
Bulgaria	6	8	0.75
Canada	3	6	0.50
**Chile**	**4**	**4**	**1.00**
China	1	3	0.33
**Colombia**	**3**	**3**	**1.00**
**All 88 cases**	**199**	**534**	**0.37**

First 15 cases out of 88

Data: Happiness in Nations (Veenhoven 2015), Table 122G

Countries in bold are illustrative of the 10 excess phenomenon

**Table 5:** Distribution of responses to a question on ‘Best-Worst possible life’ (Cantril ladder) in the years 2006–2009 on a *11*-*step* numerical scale

	0	1	2	3	4	5	6	7	8	9	10	TON ratio
Angola	2.50	10.00	8.70	16.80	20.20	20.30	10.10	5.50	3.30	0.80	0.60	0.75
**Argentina**	4.50	1.60	2.20	5.40	7.20	19.50	13.80	16.30	17.00	4.90	7.00	**1.43**
Bangladesh	0.00	2.10	16.80	8.80	21.30	28.10	9.60	4.80	5.70	1.80	0.60	0.33
Bolivia	1.20	2.90	4.20	6.70	11.90	29.50	12.70	12.90	9.40	4.10	3.90	0.95
**Brazil**	1.80	2.10	2.80	6.10	7.80	21.00	14.70	13.10	14.50	3.80	11.30	**2.97**
United Kingdom	0.80	0.50	2.30	4.20	6.70	21.40	10.90	21.50	19.90	6.60	4.60	0.70
Bulgaria	5.70	7.80	12.30	19.30	16.60	21.20	7.10	4.30	3.30	0.60	0.20	0.33
Canada	0.40	0.30	0.60	1.10	3.90	15.50	10.50	22.90	29.00	7.90	6.90	0.87
**China**	3.10	2.60	2.90	6.80	9.20	32.90	19.40	10.10	9.50	1.70	1.80	**1.06**
Czech Republic	1.00	0.00	1.00	4.40	9.30	27.50	16.00	22.90	13.20	2.60	2.00	0.77
Egypt	0.10	0.20	1.20	4.10	7.70	18.70	22.40	18.30	15.50	7.90	3.20	0.41
France	0.00	0.40	1.60	1.20	4.90	20.90	14.20	28.20	22.90	3.90	1.60	0.41
Germany	0.80	0.70	1.80	3.90	5.80	22.70	15.10	21.60	18.50	4.90	3.60	0.73
Ghana	1.00	2.60	7.40	16.40	19.50	26.20	14.10	7.50	2.10	0.60	0.30	0.50
**Guatemala**	1.00	0.40	0.20	1.20	3.60	11.60	10.20	17.40	29.00	11.40	13.60	**1.19**

Data: Happiness in Nations (Veenhoven 2015), Table 31D

Countries in bold are illustrative of the 10 excess phenomenon

Of the 833 distributions on scale 1–10 observed in 97 nations, 462 had a TON greater than 1, which is 55 %. Among these 97 nations, 23 systematically had a TON higher than 1. These were: Argentina, Bosnia Herzegovina, Brazil, Colombia, El Salvador, Guatemala, India, Indonesia, Jordan, Luxembourg, Mali, Malta, Montenegro, Morocco, Peru, Philippines, Poland, Puerto Rico, Taiwan, Tanzania, Trinidad and Tobago, Uganda, Uruguay, and Zimbabwe.

Of the 534 distributions on scale 0–10 observed in 88 nations, 199 had a TON greater than 1, which represents a percentage of 37 %. Among these 88 nations, 23 always had a TON higher than one: Belize, Brazil, Chile, Colombia, Costa Rica, Dominican Republic, Ecuador, Guatemala, Guyana, Honduras, Hong Kong, Jamaica, Macao, Nicaragua, Palestine, Peru, South Africa, Surinam, Trinidad and Tobago, Turkey, Uganda, Uzbekistan, Venezuela, and Vietnam.

Of the 48 distributions on the Cantril ladder scale, 18 had a TON higher than 1, which is 38 %. The following countries had a TON ratio of more than 1: Argentina, Brazil, China, Guatemala, Honduras, Italy, Japan, Jordan, Mexico, Pakistan, Peru, Slovakia, South Africa, Uganda, Uzbekistan, Venezuela, Vietnam, Russia, and Pakistan.

We condensed these results in six geographical areas: Africa, Latin America, North America, Asia, Europe and the Middle East. The 10 excess pattern appears in all parts of the world, but it is particularly prevalent in Latin America and the Middle East as Table 6 shows.

**Table 6:** TON distribution in parts of the world

	TON >1/total	Ten excess frequency (%)
Africa	8/11	73
Latin America	10/10	100
USA/Canada	0/2	0
Asia	7/17	42
Europe	17/40	42
Middle East	10/10	100
Total	52/90	58

Life satisfaction in 90 nations, scale 1–10, Happiness in Nations (Veenhoven 2015), Table 122F

Next to frequency, elasticity is another characteristic to be investigated; to do so, we looked at the elasticity of the phenomenon at higher levels of TON, for instance TON inferior or equal to 2. Out of the 535 surveys presented on a 1–10 scale, the number of TON superior to 2 drops to 35, which represents 6.5 % of the surveys. Out of the 833 surveys on a 0–10 scale, 153 have a TON superior to 2, which represent over 18 % of the surveys. Finally, out of the 48 distributions on the Cantril ladder, 5 countries (10 %) have a TON superior to 2 (Brazil, Italy, Pakistan, Peru and Turkey).

## Exploration of the 10 Excess

In this part, we explore possible explanations in order to understand this phenomenon. We first consider technical reasons (survey techniques, scale effect, and wording). We then investigate societal reasons before analyzing possible cultural influences.

First we will look into *technical* reasons. The phenomenon we observe might occur in particular samples or be caused by subtle differences in survey techniques, such as in the sampling of respondents, the placement of happiness in the questionnaire and the behavior of the interviewer. If so, we can expect that TON differs across surveys in the same country. We checked using the countries where different survey programs had measured happiness on 1–10 or 0–10 numerical scales. To do so, we took the example of Brazil, a country that often presents the 10 excess. We compared different surveys. The results are shown in Table 7.

**Table 7:** Comparison of surveys in Brazil in various years

Year	Measure	Characteristics	N	Survey	0	1	2	3	4	5	6	7	8	9	10	TON
2002	C-BW-c-sq-l-11-c	18+ aged, general public, Brazil	1000	What World Thinks 2002	1.80	2.10	2.80	6.10	7.80	21.00	14.70	13.10	14.50	3.80	11.30	2.97
2008	C-BW-c-sq-l-11-c	18+ aged, general public, Brazil	1353	LAPOP 2008	0.89	0.37	1.03	3.10	4.43	15.59	15.15	17.81	19.07	10.35	12.20	1.18
2010	C-BW-c-sq-l-11-c	18+ aged, general public, Brazil	2010	LAPOP 2010	0.90	0.52	0.76	1.90	3.85	14.27	14.03	18.60	23.17	8.90	13.08	1.47
2007	C-BW-c-sq-l-11-c	18+ aged, general public, Brazil	1000	PEW survey 2007	1.20	1.60	1.00	2.60	4.60	14.70	11.60	18.00	21.30	9.70	13.40	1.38
1975	C-BW-c-sq-m-11-a	16+ aged, general public, Brazil	382	Kettering Survey	1.00	2.00	2.00	5.00	8.00	21.00	19.00	14.00	14.00	5.00	9.00	1.80
1975	O-SLW-c-sq-m-11-a	16+ aged, general public, Brazil	382	Kettering Survey	1.00	1.00	2.00	3.00	5.00	13.00	11.00	17.00	18.00	12.00	16.00	1.33
2006	O-SLW-c-sq-n-10-a	18+ aged, general public, Brazil	1495	WorldValuesSurvey 5	–	1.90	0.90	1.40	2.20	10.90	8.60	12.40	23.80	13.40	24.30	1.81
1998	O-SLW-c-sq-n-10-a	18+ aged, general public, Sul, South region, Brazil	414	WorldValuesSurv 1-5	–	2.60	0.70	2.50	4.10	11.70	10.00	13.40	21.70	9.30	23.80	2.56
1998	O-SLW-c-sq-n-10-a	18+ aged, general public, Brazil	1471	WorldValuesSurv 1-5	–	3.50	2.20	3.10	3.60	14.60	7.60	11.30	16.30	9.00	28.20	3.13
1998	O-SLW-c-sq-n-10-a	18+ aged, general public, Nordeste, Brazil	268	WorldValuesSurv 1-5	–	7.10	3.70	3.70	1.90	11.50	10.00	10.00	11.20	6.70	33.80	5.04
1998	O-SLW-c-sq-n-10-a	18+ aged, general public, North West region, Brazil	523	WorldValuesSurv 1-5	–	2.50	1.80	2.80	4.00	13.90	6.90	11.90	14.80	8.90	32.00	3.60
1998	O-SLW-c-sq-n-10-a	18+ aged, general public, Minas Gerais region, Brazil	230	WorldValuesSurv 1-5	–	0.00	0.00	0.80	3.40	11.10	13.20	15.10	23.60	7.50	25.20	3.36
1998	O-SLW-c-sq-n-10-a	18+ aged, general public, Rio de Janeiro region, Brazil	190	WorldValuesSurv 1-5	–	1.80	0.90	0.00	1.40	10.30	8.60	17.60	22.40	14.30	22.70	1.59
1998	O-SLW-c-sq-n-10-a	18+ aged, general public, São Paulo region, Brazil	428	WorldValuesSurv 1-5	–	2.70	1.70	1.60	2.60	13.30	8.40	9.80	23.20	13.00	23.20	1.78
1990	O-SLW-c-sq-n-10-aa	18+ aged, general public, Brazil, 1990	1502	WorldValuesSurvey 2	–	2.98	1.29	2.33	3.72	14.31	7.39	13.11	17.11	8.81	28.24	3.21
1996	O-SLW-c-sq-n-10-aa	18+ aged, general public, Brazil, 1996	1149	WorldValuesSurvey 3	–	4.87	2.96	3.66	3.74	13.14	8.79	9.66	14.88	8.62	29.33	3.40
2007	O-SLW-c-sq-n-11-a	15+ aged, general public, Brazil, 2007	1035	GallupWorldPoll2007	0.60	0.70	1.10	1.70	2.90	9.40	10.60	16.50	24.70	9.50	22.50	2.37

Variations can be seen between regions, years, and scales but the 10 excess phenomenon is present in every survey. We also compared the variance in TON within survey programs with variance in TON across survey programs. We compared the LAPOP, What World Thinks and PEW surveys asking the same question on contentment on a 0–10 scale in the years 2000. The LAPOP presents the same survey in the years 2008 and 2010 and their difference are very small, with a variance of 0.05. The variance among the three types of surveys is much more important (0.5), which seems due to the fact that the TON is much higher in the What World Thinks survey (2.37) whereas LAPOP (1.32) and PEW (1.38) are very close.

We hypothesized that the 10 excess phenomenon is typical for short response scales and therefore occurs more often on 1–10 scales than on the 0–10. People might be less prone to go to 10 once they have imagined what 0 means versus 1 which is less extreme. This was found to be the case: the 10 excess was rather less present on a 0 to 10 scale (37 %) than on a scale from 1 to 10 (55 %). However, it still represents a high percentage of the distributions in both cases.

The 10 excess phenomenon could be more common if the positive end of the rating scale is labeled modestly, using terms such as ‘satisfied’ or ‘happy’, rather than with stronger terms such as ‘completely satisfied’ or ‘extremely happy’. To check this explanation we selected pairs of questions used in the same country in the same period, that differed only in the labeling of the extremes of the numerical response scale. The only match in terms of length of scale, period and measure type are the questions^2^ O_SLW_c_sq_n_10_a (World Values Survey, wave 1–5, 1990–2005) and O_SLU_c_sq_n_10_b (European Quality of Life Survey, wave 2003). Both address life satisfaction on a 1–10 scale in European nations between 1990 and 2005. However, whereas the first ranges from ‘dissatisfied’ to ‘satisfied’, the second one ranges from ‘very dissatisfied’ to ‘very satisfied’. As shown in Table 8, the prevalence of the 10 excess is exactly the same in both cases (36 %), so there is no difference in the only comparison case we have.

**Table 8:** Comparison of two types of surveys

	Labeling	Number of surveys for European countries	Number of surveys presenting a 10 excess	Ratio of surveys presenting the 10 excess (%)
O_SLW_c_sq_n_10_a (World Values Survey, wave 1–5, 1990–2005)	‘Dissatisfied’ to ‘satisfied’	149	54	36
O_SLU_c_sq_n_10_b (EQLS 2003)	‘Very dissatisfied’ to ‘very satisfied’	28	10	36

Yet another possibility is that the 10 excess pattern appears more on numerical scales, because the number 10 is open to more interpretations than a word like ‘satisfied’. Ideally this requires a comparison with responses scales with an equal number of verbal response options. Such cases are unfortunately not available; the longest verbal response scales provide only seven options. Therefore we compared means obtained using numerical scales to the mean scores on verbal response scales, which were later transformed to a secondary 0–10 numerical scale. To that end, we selected average values: for numerical scales, we used the average mean score given for an 11-step numeral Life Satisfaction scale (Table 122F) and for verbal scales, the average values given for 4-step scales (Table 111 C). Both sets of data were available in the ‘Happiness in Nations’ collection in the World Database of Happiness.^3^ We then assessed whether the means on the numerical response scales tended to be higher than the means obtained using verbal response scales. We repeated this analysis for the countries where 10 excess responses were observed. The differences between average scores on the numerical 1–10 scale and the verbal scale, ‘very unsatisfied to very satisfied’, that was projected on a numerical scale to see if some differences could be observed, are presented in Table 9. There are differences between the responses to the two types of scale, but they do not seem to be systematic; in our 10 excess list, some countries like Argentina or Brazil showed quite a large difference between the verbal and the numerical scale, which is indicative that this excess came from a scale effect;. However, when looking at Venezuela, Colombia and Costa Rica, the results on the two scales were the same, and in some cases, the result on the verbal scale was even higher than that on the numerical scale. When computing the difference between average scores obtained using a numerical scale and verbal scale; a 4-step numerical scale presented more excess responding than a 10 or 11 step scale. When subtracting scores on the verbal scale from the average score on the numerical scale, the difference was +0.32 in the case of countries that did not have a 10 excess, and +0.16 in the case of countries presenting a 10 excess. Therefore, the difference was even smaller in the countries presenting a TON effect. So numerical responding does not seem to explain the bias in responses to happiness questions, quite the contrary.

**Table 9:** Mean scores on pairs of questions on life satisfaction in the same country and period

Country	Average score on 0–10 numerical scale	Average score on equivalent question rated on a verbal response scale and transformed to range 0–10	Difference
Argentina	7.3	6.39	+0.91
Armenia	5	4.78	+0.22
Austria	7.6	6.7	+0.90
Belarus	5.2	5.5	−0.30
Belgium	7.3	6.85	0.45
Belize	6.6	6.64	−0.04
Bolivia	6.3	6.12	0.18
Brazil	7.5	6.6	0.90
Bulgaria	4.4	4.17	0.23
Canada	7.8	7.91	−0.11
Chile	6.7	6.49	0.21
China	6.3	6.11	0.19
Colombia	7.7	7.39	0.31
Costa Rica	8.5	7.74	0.76
Croatia	6	5.94	0.06
Average	6.65	6.4	0.25

0–10 numerical scales compared to transformed scores on a 4 step verbal response scales

Data: Happiness in Nations (Veenhoven 2015), Tables 121C and 122F

So far we have considered the 10 excess pattern in response to questions on life satisfaction and the Cantril ladder. These two questions are related to a fairly cognitive evaluation of life. Does the same pattern appear in responses to more affective toned questions? We have yet to consider questions that use ‘happiness’ as the keyword, responses to this question are mostly recorded on shorter scales with verbal response options. Data on numerical ratings of ‘happiness’ and ‘mood’ are scarce. Still the European Social Survey includes a question with ‘happiness^4^’ as the key word, the responses of which are recorded on the same 0–10 numerical scale as the question on life-satisfaction^5^ used in the same survey. This enables us to check whether the same response pattern appears for the more affectively toned question. We find a noticeable difference, 25 % of 10 excess in responses to the question on ‘satisfaction’, and only 10 % in responses to the question on ‘happiness’. Affective measures seem to be less vulnerable to the extreme responding than more cognitive measures, at least in Europe. This difference can be understood in the context of the theory that we draw on two sources of information when evaluating our life; how well we feel affectively most of the time and to what extent we perceive that life meets standards of the good life (Veenhoven 2009). In that In such a context it seems that we are better at determining how well we feel, than in judging how successful we are in meeting standards. A possible reason for this is that there are many standards for judging life and performance on these is not always clear. It is easier to rate how you feel than rate the distance to the best possible life.

A possible explanation for this 10 excess phenomenon is that *societal* factors are responsible. The wealthiest percentile of a nation report to be significantly happier than others and wealth has one of the highest correlations with happiness (Senik 2014). In this respect, a society with a high number of 10 responses might be characterized by a particularly privileged class, whose members would easily tick the top of the scale on questionnaire. Therefore, a high income inequality might create or at least inflate the 10 excess phenomenon. Latin American countries, largely represented among the countries with a 10 excess, are also among those with the highest income difference. South Africa and Hong Kong, which are present in the 10 excess list too, are also among the most unequal countries in the world. However, this explanation has many exceptions to the rule: much more equal societies frequently show a 10 excess, e.g. Luxemburg, Czech Republic, Austria, and Mali. This is confirmed by the relatively low correlation between the TON ratio and income inequality measured with the Gini coefficient: r = +.28.^6^

Next, various *cultural* factors could be involved. Happiness is valued in most societies and claiming to be very happy could be a way to obtain prestige and social acceptance. Therefore we checked social desirability. In a study among college students in 41 nations, Diener et al. (2000) assessed the degree of life satisfaction they deemed *ideal*. Ideal scores range from 19.80 (China) to 31.14 (Australia).^7^ Ideal happiness tends to be higher in 10 excess nations; e.g. in Puerto Rico (30.70), Colombia (31.12), Brazil (29.07), Peru (28.98) and Argentina (27.72). Yet the two countries with the highest ideal happiness, Australia (31.14) and Spain (31.02), are not among the countries that frequently present a 10 excess. The correlation between the ideal life satisfaction and the TON ratio is +.27,^8^ thus the valuing of happiness might be involved, but to a small extent.

Ratings on the numerical scale of happiness could be influenced by the way school performance is graded in the country. For instance, some educational systems might adopt more extreme grades, others more in the middle for a comparable exam; we call this a *grading* *culture*. For instance, a study^9^ comparing the American, British and Dutch educational systems shows that the first ones give the most top grades whereas it is nearly impossible to get a top grade in the Netherlands. The highest grade frequency is consistent in order with the TON, at least for these three examples, America having the highest frequency of ten-excess and the Netherlands the least. We miss systematic data on grading culture that would be of much interest here. However, it is possible that the grading culture itself is embedded in a wider cultural response pattern.

The 10 excess pattern observed in responses to questions about happiness can be part of a wider tendency to tick extreme response options. If so, that must manifest it must be manifested in ratings other than happiness, such as in responses to questions about perceived freedom. We checked using the item of perceived freedom in the World Values Survey.^10^ Among the 57 nations for which data is available, 41 present a 10 excess in the feeling of freedom, which represents a percentage of 71 % of TON. Thus, the TON rate is higher for life satisfaction or happiness questions. The correlation between TON for life satisfaction and the feeling of freedom is strong: +.58,^11^ which confirms the close links between life satisfaction and perceived freedom in Brulé and Veenhoven (2014). Hence, the TON excess seems to partly echo a wider response style. The 10 excess pattern is observed in countries with different cultures; however, we do see a particularly high occurrence of this pattern in Latin America and the Middle East. This echoes sparse elements in the existing literature. As noted in Sect. 1.1, Culpepper and Zimmerman (2006) have shown that Hispanic students are more prone to extreme responding on to different topics. Baron-Epel et al. (2010) showed that Arabic students were prone to choosing extreme answers, a phenomenon also highlighted in the case of Jordan by D’Iribarne (2012) [Jordan is also one of the most dramatic examples of 10 excess: in the last dataset, the percentage of 9-respondents is 8.9 and the percentage of 10 respondents is 30.4, i.e. a TON of 3.42, the second highest after Puerto Rico (3.52)]. These results seem to be in line with those previous works; the 10 excess might therefore be largely drawn from a wider cultural measurement effect.

If the 10 excess phenomenon is at least partly due to some cultural effect, scores of average happiness in nations could be inflated in the countries with the 10 excess and lower the correlation with a nation’s characteristics, such as income per head. If that is the case, this provides us with opportunities to check whether a measurement bias is involved and get an idea of the size of this bias. As such we explored the effect of three distinct *artifacts* on the 10 excess in the distribution of happiness in nations. The first artifact was to change the frequencies of 9 and 10 responses for the countries presenting a 10 excess. The second artifact was to reduce the 10 scale by combining the responses in the following way: 1–2, 3–4, 5–6, 7–8 and 9–10. The third artifact we applied was the following: we computed the ten over nine (TON) ratio for the 371 distributions on a 1–10 scale that does not present a 10 excess; the average TON was 0.64. Assuming that a unimodal distribution was the norm, we applied this ratio to countries presenting the 10 excess to remove the bias; we then computed a new percentage of 10 respondents by multiplying the number of 9 respondents by 0.64, thus obtaining a modified 10 %, which was lower than in the original data. The sum amounted to lower than 100 %, so we computed a new average with the modified percentage of ten respondents to reach 100 % by multiplying the average by (100/(100 − ((original 10) − (corrected 10)))) so the 10 excess was distributed over all the bars, respectively of their proportional weight.

The question is then whether the means of the corresponding modified distributions in nations correlate better with societal quality than the means of the unmodified distributions. In order to verify this, we examined 97 nations around the year 2000, using the following nation characteristics: buying power per capita, the human development index, government effectiveness and economic freedom. Data was drawn from Veenhoven’s (2015) ‘States of Nations’. The results are presented in Table 10. These variables explained 69 % of the variance in unmodified average happiness in nations. A variance similar or inferior could indicate that the 10 excess resides in some objective conditions, a higher variance could indicate that some measurement bias is involved.

**Table 10:** Explained variance in average happiness in 97 nations around 2005

Average happiness in nation	Explained variance (%)
No correction	69
Merge 10 step scale into 5 step scale	70
Swap 9 10 scores	71
Transform to TON 0.64	74

With and without correction for 10 excess bias

All the correlations are significant at the 0.01 level

Data: States of nations (Veenhoven 2015) variables RGDP_2005, HDI_2009, GovEffectiveness_2006, FreeEconIndex1_2005

When modified averages of happiness was used, the explained variance rose to 71 % in the case of the 9–10 swap, 70 % in the case of the merge and up to 74 % for the TON 0.64 method. So in all cases, there was a gain in explained variance, which is rather substantial in the case of the third method, as the gains of percentage in the high percentage area are hard to achieve. This seems to confirm that some measurement bias is involved in the ‘10 excess’ phenomenon. There could be other possible artifacts, e.g. by squeezing the observed distribution on this 0–10 numerical scale into a reference distribution obtained using a survey question on the same topic, in the same year, with a different response scale (De Jonge et al. 2013). The purpose of this paper is not to select the best artifact; as for now, it is sufficient to demonstrate the plausibility of bias.

## Discussion

The 10 excess phenomenon, like many response biases, does not seem to be due to one single reason; rather it is linked to several factors (social, cultural, survey techniques, etc.). Nonetheless, it seems that some factors are more influential than others. Income inequalities seem rather unsatisfactory in explaining this phenomenon. In this context, the survey techniques, the scaling, the labeling, in brief, all the survey factors seem to play a minor role. Keywords are influential to some extent: in Europe for instance, the phenomenon is less observed on happiness than on life satisfaction. A few hints tell us the grading culture might have an influence, but the grading culture might in turn be a part of a wider response pattern. The large correlations between the 10-excess rate on various subjective questions such as happiness, life satisfaction and perceived freedom seem to indicate that a wider cultural frame is involved, with systematic presence in Latin America and in the Middle East. These findings are in line with other studies (Culpepper and Zimmerman 2006; Baron-Epel et al. 2010; D’Iribarne 2012) which also mention ERB among Latin Americans and Arab respondents.

The question of whether it is an *excess* or not is of major importance. In this article, we have chosen as a starting point the unimodal distribution mostly observed in developed nations, among others. The excess in the sense used in this article is a deviation vis-à-vis this distribution we chose. This raises a tricky question of whether we should correct it or not. As Stening and Everett (1984) stated for biases in general, “ultimately each researcher will undoubtedly vary as to what action, if any, on the variety available should be taken to correct for such biases” (p. 156). The very question of whether we should correct national distributions or not is debatable, a line we did not cross in this article, as the modifications were not *corrections* but *artifacts*, used in order to see what that would imply for commonly used correlations. If we acknowledge the answer is ‘no’, which is the most convenient case, it means that we are leaving aside part of the differences in distribution and that we should at least acknowledge it in our studies. If we acknowledge the answer is ‘yes’, it means we need to choose a norm and correct the samples vis-à-vis that norm. In that case, which norm is the *right* one? The unimodal distribution of developed nations, the V shaped distribution of Jordan or a completely different distribution? Choosing one norm is convenient but might be rather challenging as there are in the field of cross-national studies rather large clusters that are largely independent (Brulé and Veenhoven 2015). These questions are philosophical, epistemological and methodological and remain largely open. The article is essentially explorative, exploring whether it is a bias or not and of a possible correction remain untouched.

This study faced several limitations and therefore there are options for future improvement. We think we have highlighted some reasons behind the 10 excess. However, the quantitative contributions to the phenomenon should be determined in future studies. To work further on this issue, more thorough statistical work is required. Moreover, our comparison of grading cultures was limited to only three countries. The grading culture seems to be implicated, but we do not know to what extent and the causality, although it is likely to be a part of a larger cultural response pattern. More systematic data in the field of cross-national comparison of grading would be very helpful for researchers dealing with response patterns. The keyword seems to play a role in Europe, but it would be very interesting to have comparable surveys for all countries, particularly in Latin America and the Middle East. The impact of social desirability has been understudied. The sparse data we have seems to indicate that this might play a role but its actual contribution is opaque as for now.

## Footnotes

1 For reasons of readability, we will present only the first 15 countries alphabetically in the tables to come, the complete results are available online. Complete dataset: http://gaelbrule.com/data/10excess/.

2 Codes used in the collection ‘Measures of Happiness’ of the World Database of Happiness (Veenhoven 2015).

3 Transformation from verbal to numeral scores were obtained using experts ratings; for instance very satisfied corresponds to a 9.3 on a 0–10 scale, satisfied to a 6.5, quite unsatisfied to a 3.7, and very unsatisfied to a 1.3 (Veenhoven, 1993: Sect. 7/3.3.2). It is then possible to obtain a value on a 0 to 10 scale from a distribution of verbal answers.

4 All in all, how happy would you say you are?

5 All things considered, how satisfied are you with your life as a whole nowadays?

6 Significant at 0.01 level.

7 Ratings were made on the 5 item Satisfaction With Life Scale (SWLS), possible scores of which range from 7 to 35.

8 Significant at 0.01 level.

9
http://www.studyinholland.nl/documentation/grading-systems-in-the-netherlands-the-united-states-and-the-united-kingdom.pdf.

10 Item 46: Some people feel they have completely free choice and control over their lives, while other people feel that what they do has no real effect on what happens to them. Please use this scale where 1 means "no choice at all" and 10 means "a great deal of choice" to indicate how much freedom of choice and control you feel you have over the way your life turns out (code one number).

11 Significant at 0.01 level.
